# A Poisson regression model for association mapping of count phenotypes

**DOI:** 10.1186/1755-8166-7-S1-O1

**Published:** 2014-01-21

**Authors:** Saurabh Ghosh, Abhishek Chakrabortty

**Affiliations:** 1 Human Genetics Unit, Indian Statistical Institute, Kolkata, India; 2 Department of Biostatistics, Harvard University, Cambridge, USA

## Background

Clinical end-point traits are often characterized in terms of quantitative precursors. For psychiatric disorders, traits such as symptom counts often serve as endophenotypes of interest for understanding the genetic basis of the clinical end-point trait. Since such traits are discrete in nature, it may not be optimal to use standard approaches such as Analysis of Variance (ANOVA) to detect association.

## Methodology

For population level data, we propose a Poisson regression approach that computes the likelihood of the count phenotype conditional on an additive allele count at a SNP. The test statistic is asymptotically distributed as chi-squares with one degree of freedom under no association between the SNP and the phenotype. For family-based data involving trios with at least one heterozygous parent at a SNP, we use a similar Poisson regression model conditional on two indicator variables: the marker allele transmitted by the heterozygous parent and the marker allele transmitted by the other parent. A one degree of freedom test based only on the coefficient of the first indicator is protected against population stratification as it tests for association in the presence of linkage. Two degrees of freedom test based on both the indicators is also a valid test for association, but is susceptible to population stratification.

## Results and conclusions

Based on extensive simulations under different genetic models, we find that for population level data, while the asymptotic tests for ANOVA yield an inflated rate of false positives, especially when there is heteroskedasticity in the distribution of the trait across the QTL genotypes, our proposed method maintains the correct size. Moreover, our method yields uniformly more power compared to ANOVA for the different genetic parameters in our simulations. For the trio design, we find that the two degrees of freedom test is more powerful than the one degree of freedom test. We applied our method to analyze externalizing symptoms, an endophenotype correlated with alcoholism using data generated in the Collaborative Study On the Genetics of Alcoholism (COGA) project. We found significant evidence of association in the class 1 alcohol dehydrogenase subunit ADH1C in the 4q22.3 region.

